# Major distinctions between the two oligopeptide permease systems of *Bacillus subtilis* with respect to signaling, development and evolutionary divergence

**DOI:** 10.1099/mic.0.001382

**Published:** 2023-09-27

**Authors:** Tasneem Bareia, Shaul Pollak, Polina Guler, Shani Puyesky, Avigdor Eldar

**Affiliations:** ^1^​ Shmunis School of Biomedicine and Cancer Research, Faculty of Life Sciences, Tel-Aviv University, Tel-Aviv, 69978, Israel; ^†^​ Present address: Department of Plant & Environmental Sciences, Faculty of Biochemistry, Weizmann Institute of Science, Rehovot, Israel; ^‡^​ Present address: Division of Microbial Ecology, Centre for Microbiology and Environmental Science, University of Vienna, Djerassiplatz 1, 1030 Vienna, Austria

**Keywords:** *Bacillus subtilis*, biofilm, oligopeptide permease, quorum sensing

## Abstract

Oligopeptide-permeases (Opps) are used by bacteria to import short peptides. In addition to their metabolic benefit, imported short peptides are used in many Gram-positive bacteria as signalling molecules of the RRNPP super-family of quorum-sensing systems, making Opps an integral part of cell–cell communication. In some Gram-positive bacteria there exist multiple Opps and the relative importance of those to RRNPP quorum sensing are not fully clear. Specifically, in *Bacillus subtilis *, the Gram-positive model species, there exist two homologous oligopeptide permeases named Opp and App. Previous work showed that the App system is mutated in lab strain 168 and its recovery partially complements an Opp mutation for several developmental processes. Yet, the nature of the impact of App on signalling and development in wild-type strains, where both permeases are active was not studied. Here we re-examine the impact of the two permease systems. We find that App has a minor contribution to biofilm formation, surfactin production and phage infection compared to the effect of Opp. This reduced effect is also reflected in its lower ability to import the signals of four different Rap-Phr RRNPP systems. Further analysis of the App system revealed that, unlike Opp, some App genes have undergone horizontal transfer, resulting in two distinct divergent alleles of this system in *B. subtilis* strains. We found that both alleles were substantially better adapted than the Opp system to import an exogenous RRNPP signal of the *Bacillus cereus* group PlcR-PapR system. In summary, we find that the App system has only a minor role in signalling but may still be crucial for the import of other peptides.

## Introduction

**Fig. 1.**
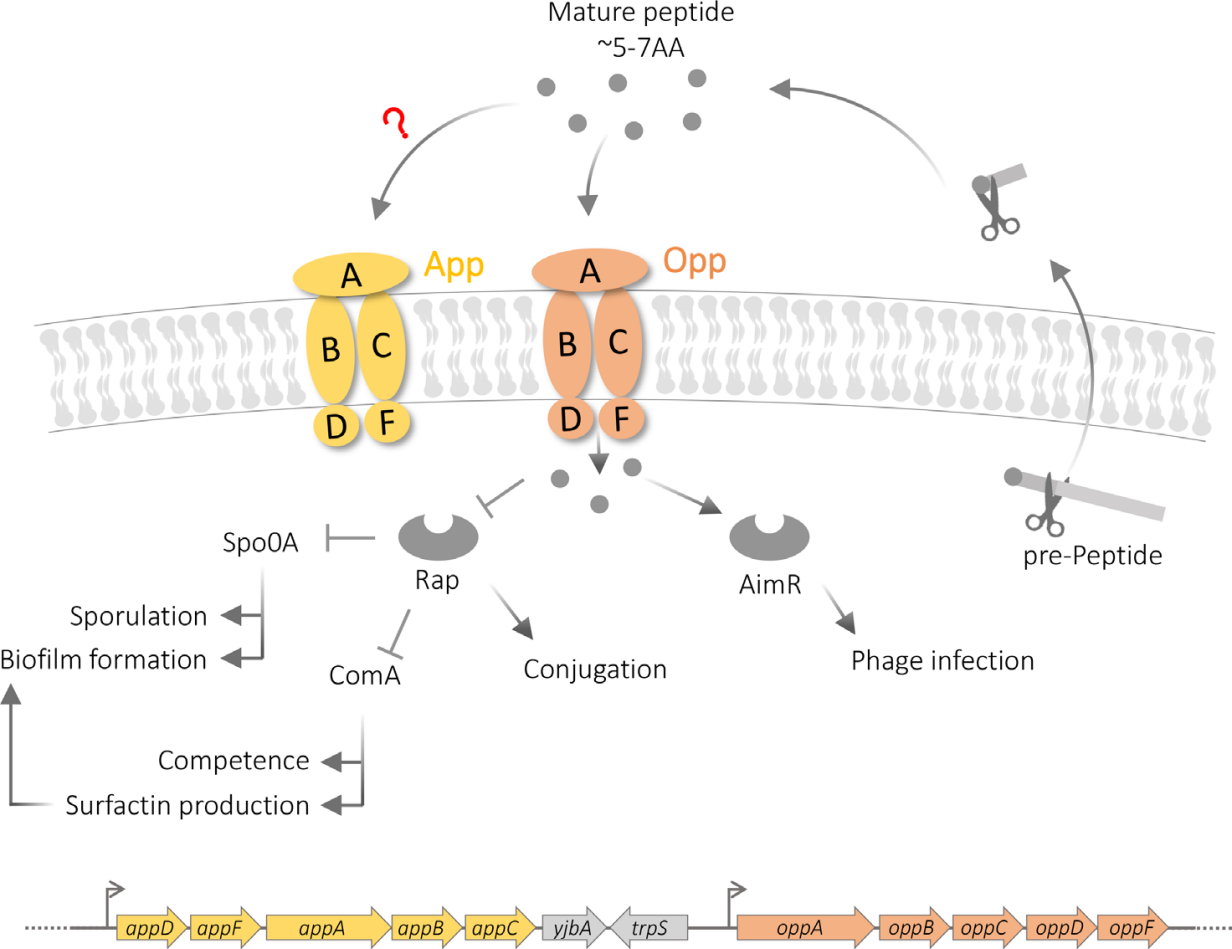
Schematic representation of App and Opp permease systems in *Bacillus subtilis *. A schematic representation of the mechanism of RRNPP family quorum-sensing systems and the oligopeptides permeases App and Opp in *B. subtilis *. Signal inhibition of Rap/AimR occurs by signal-induced conformational change of the receptor to the inactive form [8].

Oligopeptide permeases (Opp) are widespread oligopeptide transporters among various bacterial species [1]. In Gram-negative bacteria, oligopeptides are translocated by Opps from the periplasm into the cells, while in Gram-positive bacteria translocation occurs over the single membrane of the bacterium. Within the cell, oligopeptides are degraded by peptidases and utilized directly as amino acids and indirectly as nitrogen and carbon sources [2, 3]. Opp systems are ABC-transporters that hydrolyse ATP to internalize oligopeptides with length ranges from 3 up to 23 amino acids [1, 4, 5]. Each transporter system is a complex of five proteins named: OppA, OppB, OppC, OppD and OppF. Typically, all subunits are encoded by a single *opp* operon (Fig. 1). The substrate-binding protein OppA is the subunit responsible for capturing the extracellular oligopeptides. In Gram-positive bacteria, OppA is an extracellular lipoprotein that is anchored to the membrane and it is responsible for capturing oligopeptides [1]. The oligopeptides are transmitted into the cells through a channel formed by the transmembranal proteins OppB and OppC. The entire process of translocating the oligopeptides requires ATP that is hydrolysed by both intracellular subunits OppD and OppF.

In Gram-positive bacteria, short oligopeptides serve also as signalling molecules in bacterial cell–cell communication through the RRNPP family of quorum-sensing systems [6–8]. The majority of RRNPP QS systems code for a pre-pro-peptide gene adjacent to the receptor gene. The pre-pro-peptide is cleaved twice or more to form the mature signalling peptide of length 5–15 amino acids. These signalling peptides are imported into the cells using Opps. Once inside the cell, the signalling peptides interact with their cognate receptor containing a peptide-binding TPR domain. This interaction modifies bacterial gene expression, either directly or indirectly. There are multiple families of RRNPP systems. Each family shows a large diversity of peptides utilized as signalling molecules, with any given peptide often showing high specificity to its cognate receptor [9]. Through their impact on signalling, Opps are involved in the regulation of multiple bacterial traits, such as transfer of genetic elements (* Enterococcus faecalis* and *B. subtilis *) [10–12], competence (*Streptococci* and *B. subtilis *) [13, 14], virulence (* Bacillus cereus *) [15, 16], biofilm formation and sporulation (* Bacilli *) and more [4, 14, 17–19].

The model Gram-positive species, *B. subtilis* and its mobile elements, code for two families of RRNPP quorum-sensing systems. The first is the Rap-Phr system, which shows very high signal diversity and has multiple paralogous and orthogonal copies in every genome [9]. The Rap receptors of this family regulate cellular activity through protein–protein interaction and modification of several cellular targets, including Spo0A, a phosphorelay component involved in controlling sporulation and biofilm formation, and ComA, a transcription factor, which regulates competence and surfactin production [20, 21] (Fig. 1). Some members of the Rap-Phr systems control conjugation of mobile elements through interaction with specific element-encoded factors [11, 12]. A similar function is regulated by the second RRNPP family called arbitrium or AimR-AimP. Arbitrium systems are encoded by temperate *Bacillus* phages and control phage transitions between lysis and lysogeny, through direct transcriptional regulation by the AimR receptor [17, 22].

*B. subtilis* codes for two full oligopeptide permease systems named Opp and App. These systems are separately encoded by two neighbouring operons. Each operon codes for all five proteins forming the respective permease system (Fig. 1). Similarity of homologous proteins in the two systems is low and especially between the peptide-binding proteins AppA and OppA. Analysis of the peptide import preference of the two permeases, suggests that Opp shows higher specificity for shorter peptides (3-6AA) while App imports longer peptides (5-10AA) [2, 23, 24]. The overlap in permease specificity (5-6AA) matches the length of known Phr or AimP signalling peptides. As the lab strain 168 and its derivatives (PY79 and JH642) carry a frameshift mutation in the *appA* gene, the App system is inactive and therefore relatively little work has been done on it. In contrast, the Opp system, through its regulation of quorum sensing, has been shown to serve as an important system for the import of signalling peptides that control sporulation, competence, biofilm formation, surfactin production, conjugation and lysis-lysogeny decisions [4, 11, 12, 14, 17–19]. App was originally identified through a compensatory correcting mutation in *appA*, which was selected upon deletion of the *oppA* gene. Subsequent analysis suggested that App has a similar effect on sporulation and competence in *B. subtilis* lab strains 168 and its derivatives, which are deficient for biofilm formation and other traits [25–27].

While these results suggest that App and Opp are redundant in their function in *B. subtilis* quorum sensing, the extent of this redundancy and its relevance to additional traits such as biofilm formation and phage infection were not previously analysed. A recent analysis of the related species *Bacillus thuringiensis* identified two oligopeptide permease systems (named Opp and Npp). This work showed that both permeases can translocate the NprX signalling peptide of the NprR-NprX RRNPP system, but that Npp cannot translocate peptides of other RRNPP systems [28]. This further strengthens the possibility that *B. subtilis* App and Opp systems also has different roles in signalling.

Here, we explore the role of the App permease in surfactin production, biofilm formation and phage lysis/lysogeny decision as well as assaying directly the uptake of quorum-sensing peptides in a biofilm-proficient derivative of *B. subtilis *, where App is functional. We show that App permease is unable to fully compensate for Opp deletion in biofilm formation and surfactin production. Moreover, by comparing the ability of both permeases to import several peptides varied in their length and sequence, we show that the App poorly imports *B. subtilis* quorum-sensing oligopeptides including those involved in competence and sporulation. Finally, we show the existence of two genetically distinct App alleles in the *B. subtilis* group. Phylogenetic analysis suggests that these alleles were moved by horizontal gene transfer. Surprisingly, these systems have higher affinity for a *Bacillus cereus* group PapR signalling peptide than the Opp system. Therefore, our results point out functional distinctions in peptide specificity between the different systems, demonstrating that App has a minor role in RRNPP signalling and identifying an exogenous signalling peptide, which is preferentially imported through the App permease.

## Methods

### Growth media

Routine *B. subtilis* growth was performed in Luria–Bertani (LB) broth: 1 % tryptone (Difco), 0.5 % yeast extract (Difco), 0.5 % NaCl. Experiments were performed using Spizizen minimal medium (SMM; 2 g l^−1^ (NH_4_)_2_SO_4_, 14 g l^−1^ K_2_HPO_4_, 6 g l^−1^ KH_2_PO_4_, 1 g l^−1^ trisodium citrate dihydrate, 0.2 g l^−1^ MgSO_4_∙7H_2_O) [29, 30], with 0.5 % glucose as a carbon source and trace elements (125 mg l^−1^ MgCl_2_∙6H_2_O, 5.5 mg l^−1^ CaCl_2_, 13.5 mg l^−1^ FeCl_2_∙6H_2_O, 1 mg l^−1^ MnCl_2_∙4H_2_O, 1.7 mg l^−1^ ZnCl_2_, 0.43 mg l^−1^ CuCl_2_∙2H_2_O, 0.6 mg l^−1^ CoCl_2_∙6H_2_O, 0.6 mg l^−1^ Na_2_MoO_4_∙2H_2_O) [30]. When preparing plates, medium was solidified by addition of 2 % agar. Antibiotics were added (when necessary) at the following concentrations: spectinomycin: 100 µg ml^−1^, tetracycline: 10 µg ml^−1^, chloramphenicol: 5 µg ml^−1^, kanamycin: 10 µg ml^−1^, erythromycin: 3 µg ml^−1^, phleomycin: 2.7 µg ml^−1^, MLS: 3 µg ml^−1^ erythromycin +25 µg ml^−1^ lincomycin, ampicillin for *E. coli*: 100 µg ml^−1^. Isopropyl-β-d-thiogalactopyranoside (IPTG- Sigma) was added to the liquid medium when appropriate, at the concentrations indicated in the text. Sporulation promoting media DSM and was prepared as described before [31]. Biofilm growth was done on MSgg medium plates; MSgg medium contains 100 mM morpholinepropanesulfonic acid (MOPS) (pH 7), 0.5 % glycerol, 0.5 % glutamate, 5 mM potassium phosphate (pH 7), 50 µg ml^−1^ tryptophan, 50 µg ml^−1^ phenylalanine, 50 µg ml^−1^ tryptophan, 2 mM MgCl_2_, 700 µM CaCl_2_, 50 µM FeCl_3_, 50 µM MnCl_2_, 2 µM thiamine, and 1 µM ZnCl_2_ [32 ]. Media were solidified using 1.5 % agar (Difco).

### Strain and plasmid constructions

The receptors of quorum-sensing systems were integrated down-stream of constitutive promoter (*rapP*, *plcR*) or IPTG inducible ones (*rapA*, *rapC*, *rapF*). IPTG was added to a final concentration of 30 µM for the induction of P_hyperspank_-*rapA*, P_hyperspank_-*rapC* and P_hyperspank_-*rapF*. All strains expressing either App or Opp contain a full deletion of the second operon *opp* or *app,* respectively. Except for the RapP strains and the sporulation experiment strains that contain deletion of either the *appA* gene in Opp-only strain (O^+^;A^-^) or the *oppD* gene in App-only strain (O^-^;A^+^).

All strains and plasmids used in this study are listed in Table 1 and Table 2, respectively. All strains were constructed in *B. subtilis* strain 3610 plasmid-free background, unless otherwise indicated. Either a standard transformation or a Spp1 transduction method was used for genomic integration and plasmid transformation [30], and transformants were selected on plates with an appropriate antibiotic. Later genomic DNA was purified and insertion of each construct into an ectopic site in *B. subtilis* genome was verified by PCR with primers that annealed outside the homologous regions.

**Table 1.** Strain list

Name	Genotype	Preparation	Used in figure
**Strain background PY79**			
AES101	* Bacillus subtilis* PY79 wild-type	BGSC	**PY79;WT** (O^+^;A^-^) **Fig. (2a**)
AES4967	PY79; ∆*oppD::*KanR	Transformation of AES1603 into AES101	**∆*oppD*** (O^-^;A^-^) **Fig. (2a**)
AES8139	PY79; ∆*oppD::*KanR; *appA* ^3610^	Transformation of AES1603 into AES101	**∆*oppD;appA^3610^*** (O^-^;A^+^) **Fig. (2A**)
**Strain background 3610**			
AES1603	*3610;* ∆*oppD::*KanR	BGSC	
DS2569	*Bacillus subtilis NCIB3610* pBS32 cured	Kind gift from the Kearns lab	**Wildtype** (O^+^;A^+^) **Figs. (2b, 3a, 4 and S1**)
AES4959	*3610* plasmid cured; ∆*appA*::TetR	Transformation of LFH-PCR (tetR cassette) into DS2569	**∆*appA*** (O^+^;A^-^) **Fig. (2b**)
AES4720	*3610* plasmid cured; ∆*oppD::*KanR	Transformation of AES1603 into DS2569	**∆*oppD*** (O^-^;A^+^) **Fig. (2b**)
AES5309	*3610* plasmid cured; ∆*oppD::*KanR; ∆*appA*::TetR	Transformation of AES4959 into AES4720	**∆*appA*;∆*oppD*** (O^-^;A^-^) **Fig. (2b**)
AES5424	*3610* plasmid cured*; lacA*::(P*_veg_*-R0-mCherry-Mls)	Transformation of pAEC1441 into DS2569	
AES5425	*3610* plasmid cured*; lacA*::(P*_veg_*-R0-2xmTag-BFP-Mls)	Transformation of pAEC1444 into DS2569	
AES6372	*3610* plasmid cured; ∆*oppABCDF*::KanR	Transformation of LFH-PCR (BKK cassette) into DS2569	**∆*oppABCDF*** (O^-^;A^+^) **Figs. (3a, 4 and S1**)
AES7144	*3610* plasmid cured; ∆*appDFABC*::KanR	Transformation of LFH-PCR (BKK cassette) into DS2569	**∆*appDFABC*** (O^+^;A^-^) **Figs. (3a, 4 and S1**)
AES6405	*3610* plasmid cured; ∆*oppABCDF*	Transformation of pECE274 into AES6372	
AES7249	*3610* plasmid cured; ∆*oppABCDF;* ∆*appDFABC*::KanR	Transformation of LFH-PCR (BKK cassette) into AES6405	**∆*oppABCDF;* ∆*appDFABC*** (O^-^;A^-^) **Figs. (3a, 4 and S1**)
AES7376	*3610* plasmid cured*; sacA*::(P*_spoIIG1_*-3xYFP- Cm)*; lacA*::(P*_veg_*-R0-mCherry-Mls); ∆*rapA-phrA*::TetR; *amyE::*(P_hyperspank_ *-rapA-*Spec)	Lab stock	
AES7410	*3610* plasmid cured*; sacA*::(P*_spoIIG1_*-3xYFP- Cm)*; lacA*::(P*_veg_*-R0-mCherry-Mls); ∆*rapA-phrA*::TetR; *amyE::*(P_hyperspank_ *-rapA-*Spec); ∆*oppABCDF*::KanR	Transduction of AES6372 into AES7376	**RapA – App** (O^-^;A^+^) **∆*oppABCDF*** **Fig. (5e)**
AES7377	*3610* plasmid cured*; sacA*::(P*_spoIIG1_*-3xYFP- Cm)*; lacA*::(P*_veg_*-R0-2xmTag-BFP-Mls); ∆*rapA-phrA*::TetR; *amyE::*(P_hyperspank_ *-rapA-*Spec);	Lab stock	
AES7413	*3610* plasmid cured*; sacA*::(P*_spoIIG1_*-3xYFP- Cm)*; lacA*::(P*_veg_*-R0-2xmTag-BFP-Mls); ∆*rapA-phrA*::TetR; *amyE::*(P_hyperspank_ *-rapA-*Spec); ∆*appDFABC*::KanR	Transduction of AES7144 into AES7377	**RapA – Opp** (O^+^;A^-^) **∆*appDFABC*** **Fig. (5e)**
AES4654	*3610* plasmid cured*; sacA*::(P*_srfA_*-3xYFP- Cm)*; amyE::*(P*_comQXP_-rapP* ^T236N^-Spec); pECE59::P_43_-sRBS-mCherry Cm Mls;	Lab stock	
AES5514	*3610* plasmid cured*; sacA*::(P*_srfA_*-3xYFP- Cm)*; amyE::*(P*_comQXP_-rapP* ^T236N^-Spec); pECE59::P_43_-sRBS-mCherry Cm Mls; ∆*oppD::*KanR	Transduction of AES1603 into AES4654	**RapP – App** (O^-^;A^+^) **∆*oppD*** **Fig. (5d and S2)**
AES4656	*3610* plasmid cured*; sacA*::(P*_srfA_*-3xYFP- Cm)*; amyE::*(P*_comQXP_-rapP* ^T236N^-Spec); pECE59::P_43_-sRBS-mTag-2xBFP2 Cm Mls;	Lab stock	
AES5524	*3610* plasmid cured*; sacA*::(P*_srfA_*-3xYFP- Cm)*; amyE::*(P*_comQXP_-rapP* ^T236N^-Spec); pECE59::P_43_-sRBS-mTag-2xBFP2 Cm Mls; ∆*appA*::TetR	Transduction of AES4959 into AES4656	**RapP – Opp** (O^+^;A^-^) **∆a*ppA*** **Fig. (5d and S2)**
AES6975	*3610* plasmid cured*; amyE*::(P_43_-*plcR^B. thuringiensis 407^*-Spec); *sacA*::(P*_plcA_*-3xYFP-Cm); *lacA*::(P*_veg_*-R0-mCherry-Mls)	Lab stock	
AES7075	*3610* plasmid cured*; amyE*::(P_43_-*plcR^B. thuringiensis 407^*-Spec); *sacA*::(P*_plcA_*-3xYFP-Cm); *lacA*::(P*_veg_*-R0-mCherry-Mls); ∆*oppABCDF*::KanR	Transformation of AES6372 into AES6975	(O^-^; ***App^3610^***) **∆*oppABCDF*** **Fig. (8a-c and S5)**
AES6976	*3610* plasmid cured*; amyE*::(P_43_-*plcR^B. thuringiensis 407^*-Spec); *sacA*::(P*_plcA_*-3xYFP-Cm); *lacA*::(P*_veg_*-R0-2xmTag-BFP-Mls)	Lab stock	
AES7207	*3610* plasmid cured*; amyE*::(P_43_-*plcR^B. thuringiensis 407^*-Spec); *sacA*::(P*_plcA_*-3xYFP-Cm); *lacA*::(P*_veg_*-R0-2xmTag-BFP-Mls); ∆*appDFABC*::KanR	Transduction of AES7144 into AES6976	(**O*pp^3610^;*** A^-^) **∆*appDFABC*** **Fig. (8a-b)**
AES7322	*3610* plasmid cured*; amyE*::(P_43_-*plcR^B. thuringiensis 407^*-Spec); *sacA*::(P*_plcA_*-3xYFP-Cm); *lacA*::(P*_veg_*-R0-2xmTag-BFP -Mls); ∆*oppABCDF*::KanR; *appDFABC^TU-B-10T^*	Transduction of AES7248 into AES6976	(O^-^;**App^TU-B-10^**) ***oppABCDF*; App^TU-B-10^** **Fig. (8c)**
AES8170	*3610* plasmid cured*; amyE*::(P_43_-*plcR^B. thuringiensis 407^*-Spec); *sacA*::(P*_plcA_*-3xYFP-Cm); *lacA*::(P*_veg_*-R0-2xmTag-BFP-Mls); ∆*oppABCDF;* ∆*appDFABC*::KanR	Transduction of AES7249 into AES6976	(O^-^;A^-^) **∆*appDFABC;* ∆*oppABCDF;*** **Fig. S5**
AES1733	*Bacillus subtilis spizizenii TU-B-10T wild-type*	2A11T from BGSC	
AES6915	*B. subtilis spizizenii TU-B-10T;* ∆*oppABCDF*::KanR	Transformation of AES6372 into AES1733	
AES7072	*3610* plasmid cured; ∆*oppABCDF*::KanR; *appDFABC^TU-B-10T^*	Transformation of AES6915 into AES4959	
AES7248	*3610* plasmid cured; ∆*oppABCDF*::KanR; *appDFABC^TU-B-10T^*	Transformation of AES7072 into AES4959	
AES5655	*3610* plasmid cured*; amyE*::(P*_oppA_-*3xYFP-Spec)	Transformation of pAEC1471 into DS2569	**P*_oppA_*-YFP** **Fig [6]**.
AES5657	*3610* plasmid cured*; amyE*::(P*_appD_*-3xYFP-Spec)	Transformation of pAEC1473 into DS2569	**P*_appD_*-YFP** **Fig [6]**.
AES6658	*3610* plasmid cured; ∆*rapF-phrF;* ∆*rapC-phrC; amyE*::(P_hyperspank_-*rapC*-Spec); *sacA*::(P*_srfA_* 3xYFP-Cm); ∆*oppABCDF*::KanR;	Lab stock	
AES6709	*3610* plasmid cured; ∆*rapF-phrF;* ∆*rapC-phrC; amyE*::(P_hyperspank_-*rapC*-Spec); *sacA*::(P*_srfA_* 3xYFP-Cm); ∆*oppABCDF*::KanR; *lacA*::(P*_veg_*-R0-mCherry-Mls)	transduction of AES5424 into AES6658	**RapC – App** (O^-^;A^+^) **∆*oppABCDF*** **Fig. (5b)**
AES6855	*3610* plasmid cured; ∆*rapF-phrF;* ∆*rapC-phrC; amyE*::(P_hyperspank_-*rapC*-Spec); *sacA*::(P*_srfA_* 3xYFP-Cm); *lacA*::(P*_veg_*-R0-2xmTag-BFP-Mls);	Lab stock	
AES7204	*3610* plasmid cured; ∆*rapF-phrF;* ∆*rapC-phrC; amyE*::(P_hyperspank_-*rapC*-Spec); *sacA*::(P*_srfA_* 3xYFP-Cm); *lacA*::(P*_veg_*-R0-2xmTag-BFP-Mls); ∆*appDFABC*::KanR	Transduction of AES7144 into AES6855	**RapC – Opp** (O^+^;A^-^) **∆*appDFABC*** **Fig. (5b)**
AES6656	*3610* plasmid cured; ∆*rapF-phrF;* ∆*rapC-phrC; amyE*::(P_hyperspank_-*rapF*-Spec); *sacA*::(P*_srfA_* 3xYFP-Cm); ∆*oppABCDF*::KanR	Lab stock	
AES6704	*3610* plasmid cured; ∆*rapF-phrF;* ∆*rapC-phrC; amyE*::(P_hyperspank_-*rapF*-Spec); *sacA*::(P*_srfA_* 3xYFP-Cm); ∆*oppABCDF*::KanR; *lacA*::(P*_veg_*-R0-mCherry-Mls)	Transduction of AES5424 into AES6656	**RapF – App** (O^-^;A^+^) **∆*oppABCDF*** **Fig. (5c)**
AES6853	*3610* plasmid cured; ∆*rapF-phrF;* ∆*rapC-phrC; amyE*::(P_hyperspank_-*rapF*-Spec); *sacA*::(P*_srfA_* 3xYFP-Cm); *lacA*::(P*_veg_*-R0-2xmTag-BFP-Mls)	Lab stock	
AES7202	*3610* plasmid cured; ∆*rapF-phrF;* ∆*rapC-phrC; amyE*::(P_hyperspank_-*rapF*-Spec); *sacA*::(P*_srfA_* 3xYFP-Cm); *lacA*::(P*_veg_*-R0-2xmTag-BFP-Mls); ∆*appDFABC*::KanR	Transduction of AES7144 into AES6853	**RapF – Opp** (O^+^;A^-^) **∆*appDFABC*** **Fig. (5c)**
AES1605	*3610* plasmid cured*; amyE*::(P*_srfA_*-3xYFP-Spec)	Lab stock	**Wildtype** (O^+^;A^+^) **Fig. (3b)**
AES5620	*3610* plasmid cured*; amyE*::(P*_srfA_*-3xYFP-Spec); ∆*oppD::*KanR	Transformation of AES4720 into AES1605	**∆*appA*;∆*oppD*** (O^-^;A^-^) **Fig. (3b)**
AES5622	*3610* plasmid cured*; amyE*::(P*_srfA_*-3xYFP-Spec); ∆*appA::*TetR	Transformation of AES4959 into AES1605	**∆*appA*** (O^+^;A^-^) **Fig. (3b)**
AES5645	*3610* plasmid cured*; amyE*::(P*_srfA_*-3xYFP-Spec); ∆*appA::*TetR; ∆*oppD::*KanR	Transduction of AEC5309 into AES5622	**∆*oppD*** (O^-^;A^+^) **Fig. (3b)**

BGSC, Bacillus Genetic Stock Center.

**Table 2.** Plasmid list

Name	Description	Reference
pAEC277	pDL30::3xYFP-Spec (Amp)	Lab stock
pECE274	*cre* recombinase-Spec (Amp)	BGSC [34]
pAEC1471	pDL30::P*_oppA_*-3xYFP-Spec (Amp)	This study
pAEC1473	pDL30::P*_appD_*-3xYFP-Spec (Amp)	This study
pAEC1441	pAEC1226::P*_veg_*-R0-mCherry-Erm (Amp)	Lab stock
pAEC1444	pAEC1226::P*_veg_*-R0-2xmTag-BFP-Erm (Amp)	Lab stock

To prepare full deletions of *rapF-phrF*, *rapC-phrC*, *oppABCDF* and *appDEABF* operons we used a long flanking homology PCR method [33]. Primers used for each deletion are indicated in Table 3. All operons were replaced by a BKK cassette, which includes a gene for kanamycin resistance and the lox sites [34]. To generate a mutant deleted for both permeases, we removed the resistance gene from single-deletion strain using Cre recombinase expressed on the plasmid pECE274 and then integrated the second deletion.

**Table 3.** Primer list

Primer name		Sequence	Used for
OppA del P1	PTB155	TCCGCTCAAGCTCGCCGAT	LFH-PCR (BKK cassette) *opp operon* deletion
Opp BKK P2	PTB478	GGTATCCTGCCTTTCCTCCCTCCCTAATAATAATTTTCAGCTCCTTACGGC	
Opp BKK P3	PTB479	TAGAGAGAGCACAGATACGGCGCATGATTCATCAATCCTTCAAGAGAT	
Opp del P4	PTB158	TTGCAGCACAAGCCAGAGCGC	
BKK R	PSOB229	CGCCGTATCTGTGCTCTCTCTA	*opp*, *app* operons deletion
BKK F	PSOB230	GAGGGAGGAAAGGCAGGATACC	*opp*, *app*, *rapC-phrC* and *rapF-phrF* deletions
App operon del P1	PTB437	ACCTGACGAAGAATGTGATTTGG	LFH-PCR (BKK cassette) *app* operon deletion
App BKK P2	PTB522	GGTATCCTGCCTTTCCTCCCTCTCAAGAGAAAGCAATCCCTTTATTCAG	
App BKK P3	PTB523	TAGAGAGAGCACAGATACGGCGAAAGCAGGTCTCCTTTAGTCAGA	
App operon del P4	PTB440	GCAGAAGTCGTAGTGAACGCATT	
Popp **EcorI** F	PTB427	TTACT **GAATTC** AGTTCGGTCAGCTGCTCC	Cloning of pAEC1471; amplifying *oppA* promoter
Popp **NheI** R	PTB428	ATTCG **GCTAGC** GTTTTTTCATATTCGCAAACCCCC	
PappD **EcorI** F	PTB429	ATGCT **GAATTC** CGGCAAAGGATATCAGCC	Cloning of pAEC1473; amplifying *appD* promoter
PappD **BamHI** R	PTB430	ATGCG **GGATCC** TTCACTTCTAAAAGTGTGCTCATG	

LFH-PCR, Long flanking homology PCR.

The replacement of *B. subtilis* 3610 *app* operon with that of *spizizenii* TU-B-10T isolate was achieved using multiple transformations. First, deletion cassette of *opp* operon was transferred into *B. subtilis spizizenii* TU-B-10T wild-type strain (AES1733) using the LFH-PCR method [33]. Then, genomic DNA of the constructed strain AES6915, *spizizenii* TU-B-10T with *opp* deletion, was transferred into *B. subtilis* 3610 carrying *appA*::tetR (AES4959). Later, we screened for colonies that grew on kanamycin and did not grow on tetracycline plates, in which the *appA*::tetR was replaced and *opp* operon was deleted. Finally, this strain (AES7072) was backcrossed into AES4959, positive colonies screened again, and *app* operon was validated by sequencing to generate the final strain AES7248.

To generate the plasmids pAEC1471 and pAEC1473, we amplified the regions upstream of the genes *oppA* and *appD*, respectively. The primers used for each PCR reaction are indicated in the primer list. The amplified fragments and pAEC277 were then digested with the appropriate restriction enzymes (indicated on the primer) and then we ligated the fragments. The final constructs include each promoter followed by three *yfp* genes: P*_oppA_-*3 ×*yfp* and P*_appD_-*3 ×*yfp*.

### Biofilm assay

Cells were grown in SMM until optical density of OD_600_ ~0.1, then were diluted up to 0.01. MSgg plates were prepared by adding 1.5 % agar (Difco), then dried for 20 min in a laminar flow cabinet. Then, 5µ drop of the diluted bacteria was added in the middle of the dried plate, then the plate was re-dried for an additional 20 min. The plates were incubated at 30 °C for 5 days before the pictures were taken.

### Phage infection

Overnight culture grown in LB of the appropriate strain was diluted by a factor of 1 : 100 into fresh containing 0.1 mM MnCl_2_ and 5 mM MgCl_2_. The strains were then grown at 37 °C with shaking at 220 r.p.m. Upon reaching an of OD_600_=0.3, the strains infected with free ɸ3T at m.o.i.=0.1 and supplemented with 10 µM of the synthetic peptide SAIRGA. Optical density measurements at a wavelength of 600 nm were performed in a 96-well plate using a plate reader (PerkinElmer VICTOR Nivo 622 Multimode Plate Reader).

### Flow-cytometry analysis

Flow cytometry was performed to quantify gene expression at the single-cell level, using a Beckman-Coulter Gallios flow-cytometer equipped with four lasers (405 nm, 488 nm co-linear with 561 nm, 638 nm). The emission filters used were as follows: BFP – 450/50, YFP – 525/40, mCherry – 620/30. Two methods were used to distinguish between co-cultured cells. Strains coding for *rapP* where transformed by plasmids carrying either an mCherry or mTag2-BFP genes under a constitutive promoter. For the rest of the strains, the same constructs were integrated into the bacterial genome at the *lacA* locus. YFP levels were measured relative to a set voltage, which was approximately set such that a value of 1 will be given to autofluorescence of strain PY79 in SMM medium.

### Growth protocols

Cells were grown to OD_600_ <0.1, in SMM medium containing trace elements and glucose, then diluted by a factor of 10^6^ or 10^7^ into fresh SMM medium and grown for about 16 h in exponential phase. In co-culture gene-expression experiments, each strain was grown from a single colony in SMM to OD_600_ <0.1 or diluted to 0.1 prior to strain mixing. The two strains were mixed at equal volumes and then diluted by a factor of 10^6^ or 10^7^ in fresh SMM medium. Samples were taken from cultures at several time-points. At each time point OD_600nm_ was measured using a spectrophotometer and fluorescence was measured using a flow cytometry.

### Addition of external peptide

The synthetic peptides hexa-PhrP (ADRAAT), penta-PhrP (DRAAT), penta-PhrC (ERGMT), penta-PhrF (QRGMI), penta-PapR (LPFEF) and penta-PhrA (ARNQT) were purchased from GL Biochem (Shanghai, China) at >98 % purity, and a synthetic hepta-PapR peptide (ADLPFEF) was purchased from BLAVATNIK CENTER for Drug Discovery (Tel-Aviv, Israel) at purity of >95 %. And the SAIRGA hexa-peptide was purchased from Peptide 2.0 (Chantilly, VA, USA) at >96 % purity. Lyophilized peptides were resuspended with sterile distilled deionized water (sDDW) to prepare 10 mM aliquots. All receivers were grown in minimal medium (SMM), until they reached a specific optical density as indicated in the captions. Then, different concentrations of the appropriate signalling peptide were added, and 3 h later, YFP expression levels were measured using flow cytometry.

## Results

### A general scheme for comparing the impact of the two oligopeptide-permease systems

In order to phenotypically examine the role of the App and Opp permeases in biofilm-forming strain, we utilized the biofilm-forming variant of the wild strain 3610, which lacks the plasmid pBS32 [35]. This variant has been shown to be a proficient biofilm former and to have an enhanced competence due to the lack of plasmid-related repressors of competence [31, 35]. We used this background strain to construct four allelic variants; a wild-type strain (designated as O^+^;A^+^), full deletion mutants of the App (Opp-only; O^+^;A^-^) and Opp (App-only; O^-^;A^+^) operons and a mutant deleted for both permeases (designated as O^-^;A^-^). We also constructed partial deletions of the *appA* or *oppD* for comparison with previous work on sporulation [2].

### App and Opp permeases affect sporulation differently in the biofilm-forming strain and the lab strain PY79

First, we wanted to test for any compensatory interactions between the two permease systems during sporulation, which was previously studied in lab strain JH642 (a derivative of strain 168) [2]. To mimic the strains used previously, we used single-gene deletions, ∆*appA* or ∆*oppD*, for generating the mentioned four allelic variants: WT, (Opp-only; O^+^;A^-^), (App-only; O^-^;A^+^) and a strain deleted in both genes (O^-^;A^-^) (see strains construction for details). We studied sporulation in Difco Sporulation Medium (DSM), as was previously described [2]. The sporulation efficiency decreased by a factor of 30 (18–48, upper and lower estimates, logarithmic standard error) when *oppD* was deleted in the strain PY79 (*t*-test, *n*>3, *P*=0.0075) and was restored to near wild-type level by restoring App activity, in agreement with previous analysis in the lab strain JH642 [2] (Fig. 3a). In contrast, the two equivalent mutants, strains Δ*appA* (O^+^;A^-^) and Δ*appA;*Δ*oppD* (O^-^;A^-^), in 3610 background differed only by a factor of 1.6 (1.5–1.8, upper and lower estimates) (Fig. 3b). This suggests that the impact of oligopeptide uptake in the wild-isolate 3610 and sporulation medium is limited, impeding the comparison between the two permeases in this strain.

**Fig. 2.**
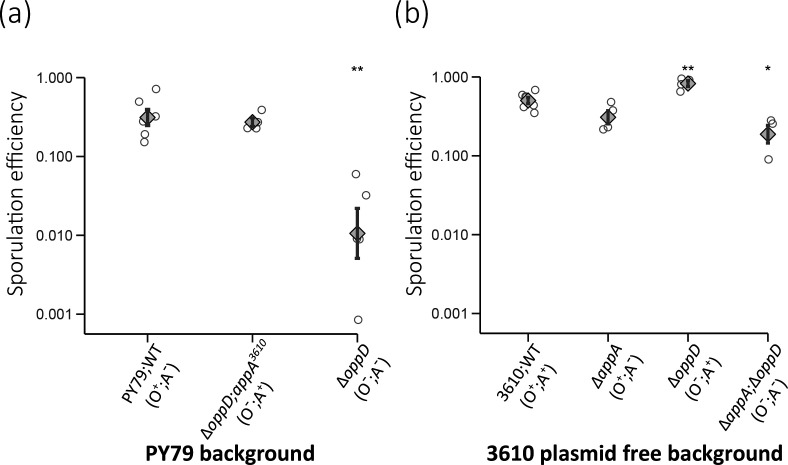
Sporulation efficiency in the background of the biofilm-forming strain and lab strain PY79. Sporulation efficiency on a logarithmic scale for different mutants. (**a**) shows results for strains in PY79 background: WT (O^+^;A^-^, AES101), *ΔoppD* (O^-^;A^-^, AES4967) and *ΔoppD;appA^3610^* (O^-^;A^+^, AES8139). (**b**) shows results for strains in 3610 plasmid-free background: WT (O^+^;A^-^, DS2569), *ΔappA* (O^+^;A^-^, AES4959), *ΔoppD* (O^-^;A^+^, AES4720) and *ΔoppD;ΔappA* (O^-^;A^-^, AES5309). Mutants are studied in each genetic background, as shown above. All strains were cultivated in Difco Sporulation Medium (DSM). Sporulation efficiency was examined for each strain for at least four independent biological repeats performed on different days. The annotation *appA* ^3610^ indicates that the *appA* locus of strain PY79 has been replaced by the *appA* locus of strain 3610. Grey rhombuses represent the mean of sporulation efficiency and error bars represent the standard error of the mean. Asterisks mark results of strains whose sporulation efficiency is statistically different from the parental background (*t*-test, * 0.01§amp;lt;p§amp;lt;0.05 , ** p§amp;lt;0.01). Note the difference in the *y*-axis between the two graphs.

### The App system does not fully compensate for deletion of the Opp system in biofilm formation and surfactin production

Biofilm formation in *B. subtilis* depends on the expression of exopolysaccharides (EPS) and the surfactant surfactin. EPS is the main compound of the extracellular matrix, and its production is regulated by Spo0A [32, 36]. surfactin is known to play a key role in forming the aerial structures of *B. subtilis* biofilm and is regulated by ComA [32]. Both ComA and Spo0A are dependent on the activity of multiple Rap-Phr quorum-sensing systems [9, 37].

To test how both permeases affect biofilm formation, we used the above four strains: wild-type strain (O^+^;A^+^), App mutant (Opp-only; O^+^;A^-^) and Opp mutant (App-only; O^-^;A^+^) and a mutant deleted for both permeases (O^-^;A^-^). To prevent any possible interaction between specific proteins from the two systems, we used deletion alleles of the whole *app* or *opp* operons (Methods). The strains were plated on MSgg plates, a biofilm-promoting medium solidified with 1.5 % agar, and then were incubated in 30 °C for 5 days (Methods) (Fig. 4a). We find that the biofilm phenotype of the Opp-only strain (O^+^;A^-^) is similar to that of wild-type (O^+^;A^+^). In contrast, the App-only strain (O^-^;A^+^) is barely able to form the biofilm and is clearly deficient in producing the aerial structures observed in the wild-type and Opp-only strains. Finally, the mutant deleted for both permeases (O^-^;A^-^) is completely unable to form any aerial structures showing a very flat colony (Fig. 4a). These results demonstrate the critical role that Opp plays in biofilm formation, the limited role of the App permease system and its inability to fully compensate for the lack of Opp in biofilm formation.

**Fig. 3.**
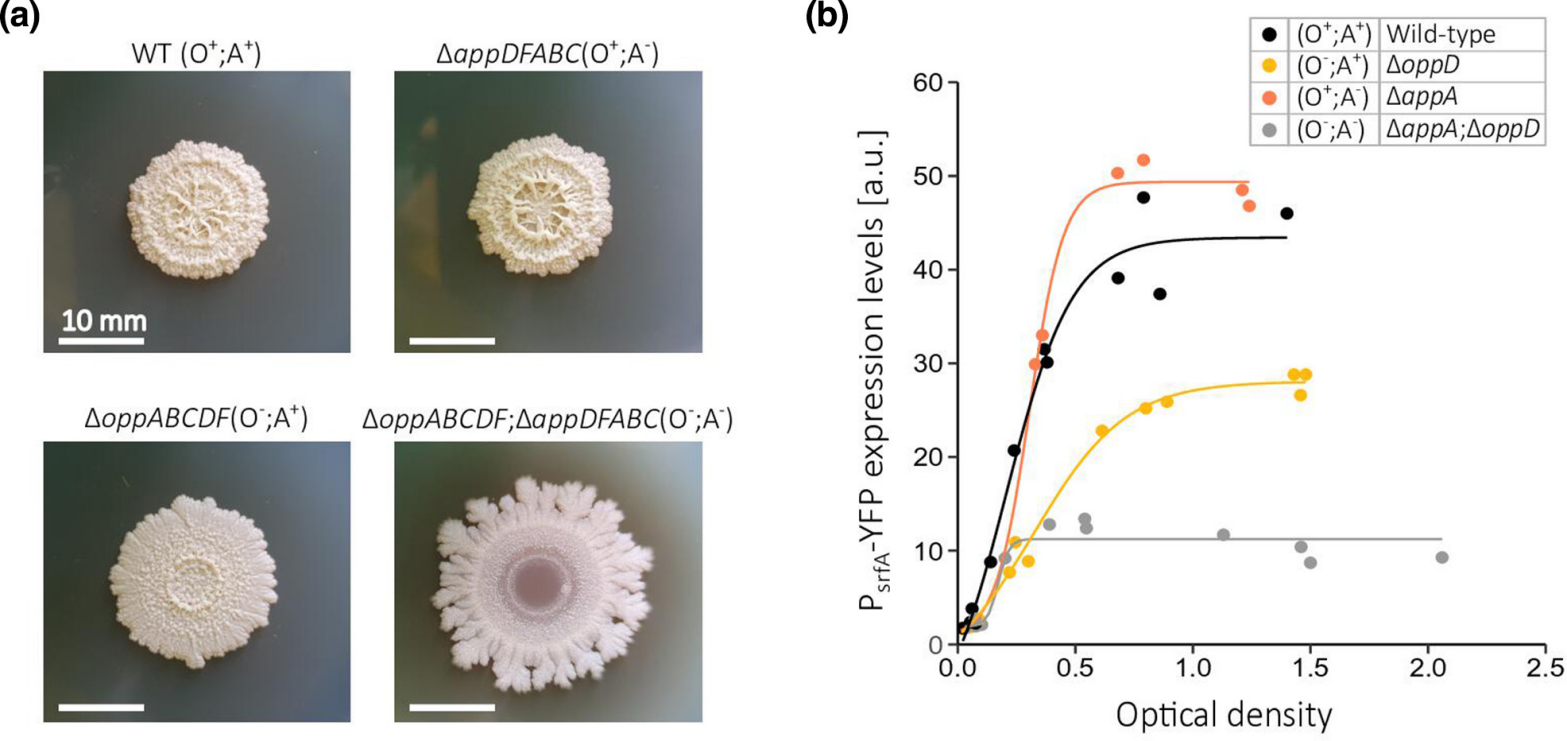
App permease has negligible impact on biofilm formation and surfactin production. (**a**) Qualitative differences in the shape of biofilm colonies of *B. subtilis* 3610 plasmid-free strains: a wild-type expressing App and Opp (O^+^;A^+^, DS2569), Opp-only strain (O^+^;A^-^, AES7144) with full deletion of *app* operon, App-only strain (O^-^;A^+^, AES6372) with full deletion of *opp* operon and a mutant deleted in both operon (O^-^;A^-^, AES7249). The strains were plated on biofilm promoting minimal medium MSgg, then were incubated for 5 days at 30 °C before the pictures were taken. (**b**) Shows YFP expression levels of the reporter P*_srfA_-*3 ×*yfp* for wild-type strain (O^+^;A^+^, AES1605, black), an *appA* null-mutant expressing Opp-only (O^+^;A^-^, AES5622, orange), an *oppD* null-mutant expressing App-only (O^-^;A^+^, AES5645, yellow) and an *appA* and *oppD* mutant strain (O^-^;A^-^, AES5620, grey) all in the background of the biofilm former 3610 isolate, as a function of their optical density. The strains were grown as pure cultures in minimal medium SMM and YFP expression levels for single cells were measured using the flow cytometry. Data was collected for three independent biological repeats.

**Fig. 4.**
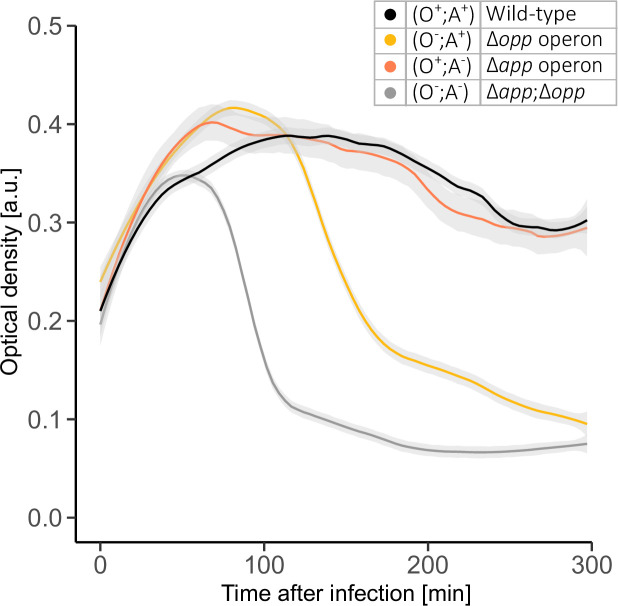
The impact of oligopeptide permeases on phage infection in the presence of an arbitrium lysogenization signal. Growth curves of different variants of *B. subtilis* 3610 plasmid-free strain: a wild-type expressing App and Opp (O^+^;A^+^, DS2569, black), Opp-only strain (O^+^;A^-^, AES7144, orange) with full deletion of *app* operon, App-only strain (O^-^;A^+^, AES6372, yellow) with full deletion of *opp* operon and a mutant deleted in both operon (O^-^;A^-^, AES7249, grey). All infected with ɸ3T at m.o.i.=0.1 in LB in the presence of 10 µM of the synthetic peptide SAIRGA. Growth curves are represented as the mean optical density (dark colour) and its error (light grey) as a function of time (minutes) taken using a plate-reader. Arbitrium peptide was added at time 0 at an OD_600_=0.3. Results are the average of three technical repeats. See additional biological repeats in Fig. S1, available in the online version of this article.

We next assayed the impact of the permease systems specifically on surfactin production. The promoter of the operon responsible for surfactin production in *B. subtilis *, P*_srfA_*, fused to three *yfp* genes (P*_srfA_*-3 ×*yfp*) was integrated into the previously mentioned strains [31, 38, 39]. Then, we monitored YFP expression levels for cells growing in minimal medium using flow cytometry. In agreement with the biofilm phenotypes, the Opp-expressing strain (O^+^;A^-^) had similar expression levels to that of the WT (O^+^;A^+^), while App-only strain (O^-^;A^+^) levels were significantly lower (Fig. 4b), but still higher than those of the mutant deleted for both permeases. These results indicate that App has a minor effect on surfactin production, while Opp is strictly necessary to reach WT expression levels.

### The App system has a limited ability to replace the Opp system during arbitrium-dependent phage infection

Previous phenotypes (biofilm formation, surfactant production) are linked with the activity of Rap-Phr systems, suggesting that App has a reduced affinity to Phr peptides. We wondered whether App is capable of transporting the signalling molecules of arbitrium, the second RRNPP system found in *B. subtilis* phages. The *B. subtilis* phage φ3T utilizes an arbitrium system with the small peptide SAIRGA to control the lysis-lysogeny and induction decisions [17, 22]. Increased signal concentration tilts the decision towards lysogeny. To further examine the impact of App and Opp on quorum-sensing-dependent activation of φ3T horizontal transfer, we utilized a plate reader to examine the phage infection dynamics by monitoring its impact on optical density during infection of the different variants in Lysogeny Broth (LB) medium. In accordance with a previous work [17], the WT (O^+^;A^+^) cells did not lyse when sufficient amounts of the peptide was added (Fig. 4, Fig. S1) (Methods). A similar behaviour was observed in the Opp-only strain (O^+^;A^-^). The App-only strain showed a marked reduction in optical density upon addition of the peptide, suggesting limited import of the peptide by the App system. The strain deleted for both permeases showed a similar but somewhat stronger response, suggesting that low levels of arbitrium peptides are imported through the App system.

### The App system has a limited effect on signaling by multiple Phr signals

Our results so far suggest that the App system is limited in its ability to compensate for the Opp system in Rap-Phr signaling-dependent traits, such as biofilm formation and surfactin production. To further examine the role of App and Opp in Rap-Phr signalling, we directly studied the differences between the App and Opp permeases by monitoring their ability to uptake several signalling peptides over a wide range of concentrations.

**Fig. 5.**
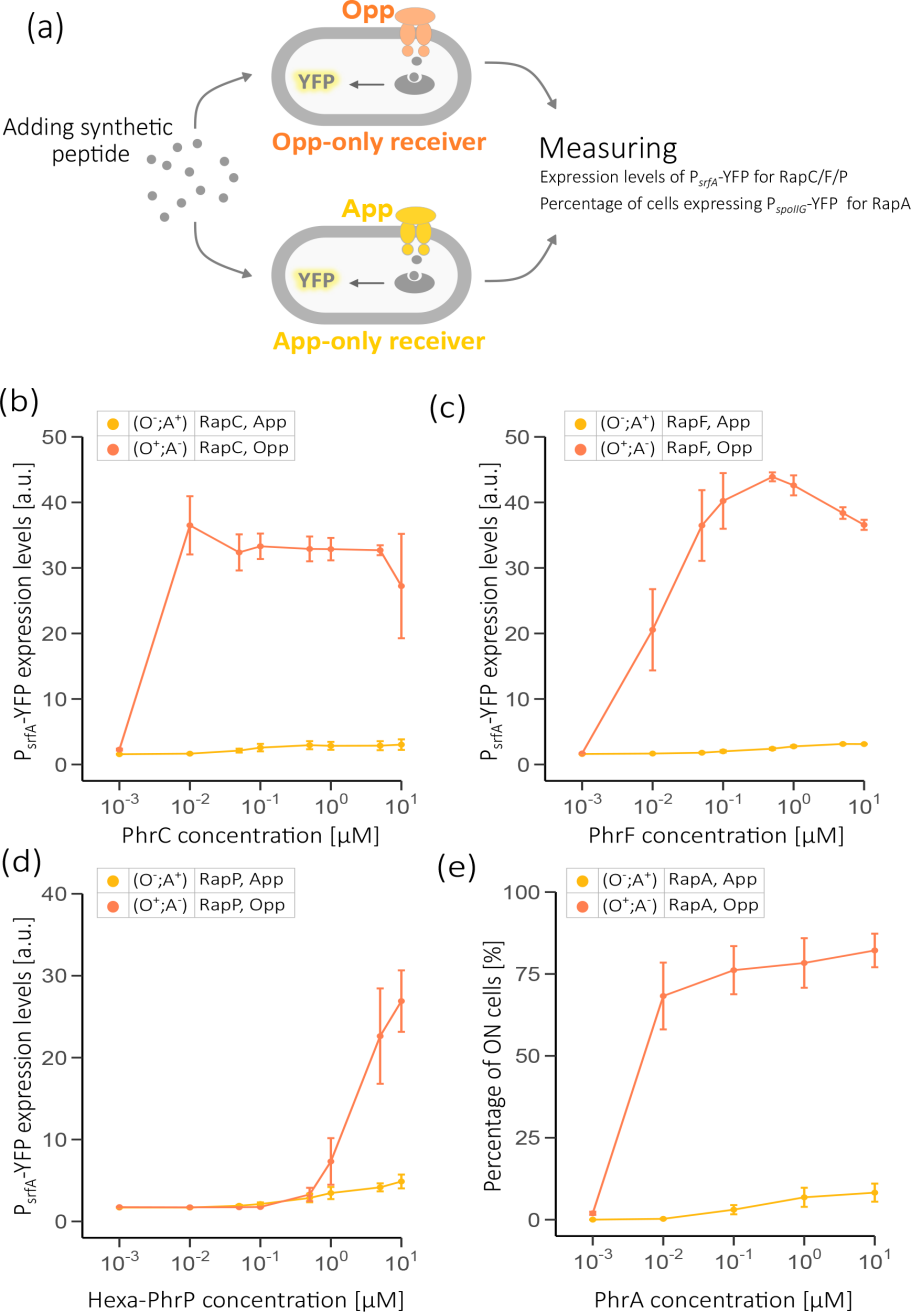
App-only strain poorly responds to extracellularly added peptides. (**a**) Schematic representation of the co-cultured receivers, each strain either expresses App-only (O^-^;A^+^) or Opp-only (O^+^;A^-^) in yellow and orange, respectively. (**b–d**) The expression levels of P*_srfA_*-3 ×*yfp* as a function of peptide concentrations for strains expressing the Rap only. In (**b**) RapC strains (O^-^;A^+^, AES6709 and O^+^;A^-^, AES7204) with PhrC peptide (ERGMT), in (**c**) RapF strains (O^-^;A^+^, AES6704 and O^+^;A^-^, AES7202) with PhrF peptide (QRGMI) and in (**d**) RapP strains (O^-^;A^+^, AES5514 and O^+^;A^-^, AES5524) with PheP Hexapeptide (ADRAAT). (**e**) The percentage of ON cells for RapA strains (O^-^;A^+^, AES7410 and O^+^;A^-^, AES7413) as a function of PhrA concentration. ON cells are determined as the cells that have expressed P*_spoIIG_-*3 ×*yfp* 10-fold higher than background. Each signalling peptide concentration was added separately at the same optical density of ~0.1 for cells growing in SMM. YFP levels were measured for single cells using flow cytometry 3 h after the peptide was added to co-culture of both strains, App and Opp. Each dot represents the mean (±sem) of at least three independent biological repeats measured in different days.

To this aim, *B. subtilis* endogenous receptor genes *rapA*, *rapC*, *rapF* and *rapP* controlled by constitutive promoters were integrated into either Opp-only (O^+^;A^-^) or App-only (O^-^;A^+^) expressing strains which lack the endogenous *rap-phr* copy (Methods). Importantly, the uptake rate of the peptide affects both its intracellular and extracellular concentration [39, 40]. Therefore, to ensure that the two single-permease expressing strains, Opp (O^+^;A^-^) and App (O^-^;A^+^), share the same level of extracellular peptide, we measured their activity using a co-culture assay (Fig. 5). We used constitutive fluorescent proteins RFP and BFP to distinguish between the strain using flow cytometry. Co-cultures were grown in SMM minimal medium, lacking other oligopeptides that may disturb the transfer of the Phr peptides through the permeases [40]. Various concentrations of the appropriate synthetic peptide (PhrC – ERGMT; PhrF – QRGMI; PhrP – DRAAT) were added extracellularly. In the case of RapP we also added the hexapeptide (ADRAAT), as RapP was shown to respond to it as well [31]. We measured YFP expression levels for each strain, 3 h following the addition of the signalling peptides. Notably, in our conditions, both strains [Opp (O^+^;A^-^) and App (O^-^;A^+^)] had a similar growth rate in monoculture and in co-culture.

RapC, RapF and RapP regulate the response regulator ComA, which activates the promoter *srfA* (41 ). We used the P*_srfA_*-3 ×*yfp* transcriptional reporter to follow the response to extracellularly added peptides [31]. This response was uniform in the population and its mean level was dependent on signalling level. Our results show that YFP levels of the App-only strains (O^-^;A^+^) marginally increased with increasing peptide concentrations, remaining similar to that of background levels. This was especially true for PhrC and PhrF, while for the two PhrP peptide variants a modest increase was recorded for peptide concentrations higher than 1 µM (Figs 2a-c and S2). In contrast, YFP expression levels were strongly induced in the Opp-only strains (O^+^;A^-^) when each signaling-peptide was added to the relevant strain (Fig. 2a-c). For RapP, half-maximal levels of expression were achieved only at peptide concentrations>1 µM. In contrast, in both PhrC and PhrF, YFP rises to half of its maximal level already at concentrations <10 nM.

RapA indirectly represses the activity of the response regulator Spo0A, the master regulator of sporulation and biofilm formation [41]. To monitor the import of PhrA, we used a sporulation-inducing medium (SMM minimal medium with a very low glucose concentration). We used a strain expressing RapA and the reporter gene for early sporulation P*_spoIIG_-*3 ×*yfp* to monitor the percentage of cells which activate sporulation as a function of PhrA peptide levels [9, 42, 43]. The same co-culture procedure was repeated (Methods). Similarly to the above results, our results show that the percentage of cells with high YFP levels increase as the PhrA concentration increases in the Opp-only strain (O^+^;A^-^) (Fig. 2e), reaching ~20 % at 10 µM. On the other hand, in the App-only strain (O^-^;A^+^) the fraction of sporulating cells at maximal concentration was 0.2 %. Altogether, these results suggest that the App permease has a strongly reduced ability to import the tested endogenous signalling peptides.

### Both permeases are expressed in minimal medium

**Fig. 6.**
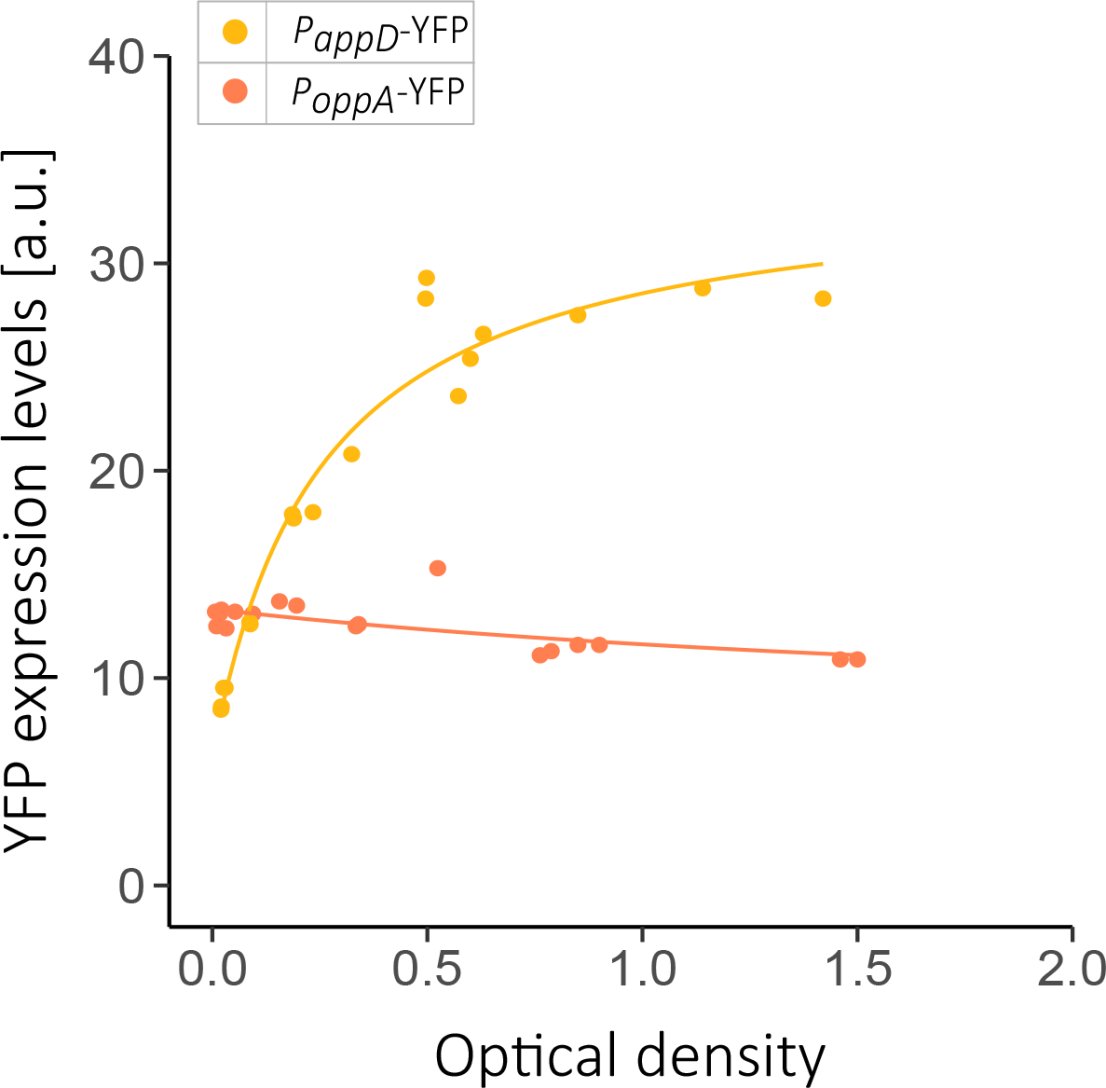
Both App and Opp are expressed during growth in minimal medium. YFP expression levels of the reporters P*_oppA_-*3 ×*yfp* (AES5655) and P*_appD_-*3 ×*yfp* (AES5657) as a function for their optical density in *B. subtilis* 3610 plasmid-free background. YFP expression levels for single cells were measured using the flow cytometry and the optical densities were measured using spectrophotometer at a wavelength of 600 nm. Each dot represents one measurement at a specific time point. The data was collected independently for four biological repeats performed on different days.

The lack of response to signalling in App-only strains in the previous experiment (Fig. 5) may stem from either the inability of App to import the signal or from low expression levels of App under the conditions used. To monitor the latter possibility, we measured the *app* and *opp* operon gene expression under growth in minimal medium. It was previously reported that the expression of the *app* promoter showed a sharp increase only in the beginning of the stationary phase, while the *opp* promoter was expressed already 90 min prior to the stationary phase in Schaeffer’s sporulation medium [44]. To examine if both permeases are expressed in our conditions, we used an *yfp* reporter gene fused to the promoter of each operon. *B. subtilis* 3610 plasmid free containing either the construct P*_oppA_-*3 ×*yfp* or P*_appD_-*3 ×*yfp* were grown in Spizizen minimal medium (SMM, Methods). We monitored the expression levels and the optical density of each strain for several time points. YFP expression levels of both permeases were significantly higher than that of the background during the exponential phase, which indicates that both permeases are expressed under our conditions. Interestingly, and in accordance with previous observation, *opp* expression seems to be constant, while *app* expression increases with cell density (Fig. 6).

### The App system has two distinct allelic variants in *B. subtilis* strains

**Fig. 7.**
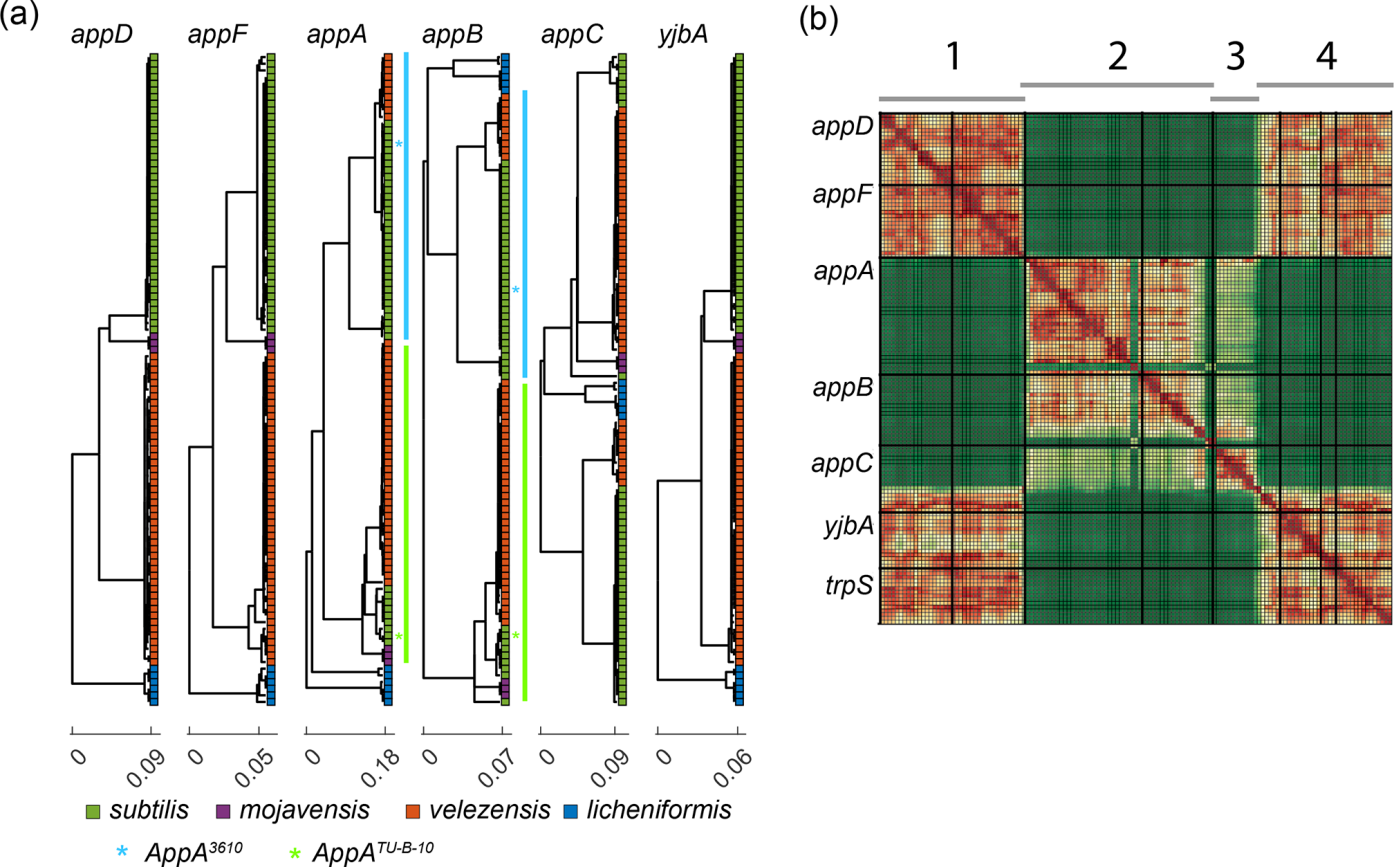
AppAB has two major variant alleles, which have undergone horizontal gene transfer. (**a**) A phylogenetic tree of the App locus protein based on 98 genomes of the *B. subtilis* group. The leaves of each tree are coloured by their specific species (see the legend below the graphs). The phylogenetic tree of AppA,B,C does not correspond to the species phylogeny. Two major alleles are indicated in AppA,B (marked by cyan and light green) with an asterisk of the same colour marking the position of strains 3610 (cyan) and TU-B-10 (light green). Phylogenetic distance is shown on the *x* axis of each graph. *yjbA* is the gene immediately downstream of the *app* locus (Fig. 1). (**b**) The heatmap shows the similarity between phylogenetic graphs taken on the DNA sequence of aligned App loci of the same 98 strains shown in (**a**). The locus was divided into regions of 50 bp windows, and a phylogenetic tree was calculated for each window. Tree similarity was calculated using the Ktreedist method [45]. Colours indicate the extent of similarity (see colorbar). Genes are marked on the *y* axis, while four regions of similarity are marked on the *x*-axis (top).

Our results so far suggest that AppA has a minor impact on signalling in strain 3610 derivatives. The high diversity of bacterial signalling may suggest that this may not be the case in other *Bacillus* strains. To further study the relation between App and Opp, we therefore characterized their presence and diversity in other strains from the *B. subtilis* group of species. We compared the patterns of diversity of the *appA* and *oppA* genes between 98 different strains of the *B. subtilis* group of species. We found that all strains encoded both the Opp and App systems, implying that they belong to the core genome of this group. In agreement with this suggestion, the Opp system shows a phylogenetic pattern highly similar to that obtained from the whole core-genome phylogenetic tree where all strains of a given species are monophyletic (Fig. S3). Surprisingly, we found that the App system displayed a pattern of allelic variation that was not congruent with the phylogenetic tree of the core genome. Specifically, we find that *B. subtilis* strains contain one of two significantly different alleles of *appAB*. A phylogenetic analysis of the *app* operon points to a phylogenetic tree, which is non-congruent with the species tree in *appA,B,C* (Fig. 7a).

To better understand this allelic variation, we performed a detailed analysis of the phylogenetic pattern of the *app* operon. We ran a moving window of 500 bp over the sequence in jumps of 50 bp and constructed phylogenetic trees for each sequence window. We then used minimal-tree distance metric (Ktreedist [45]) to compare the phylogenetic trees of each segment (Fig. 7b). We find that *appD* and *appF* showed a phylogenetic pattern congruent with the species tree, and so did the *yjbA* gene immediately downstream of the App operon. In contrast, windows within *appA* and *appB* had a highly similar tree which differed dramatically from the core genome phylogeny while *appC* had an intermediate phylogeny. This pattern suggests that the allelic diversity is focused on the *appAB* fragment, and that one allele can replace the other by recombination over the flanking genes, which may occur within the *appC* gene and immediately downstream of the *appA* gene.

### The exogenous signaling peptide PapR is efficiently imported by the two genetically distinct App permease allele

Our results so far indicate that App has a limited activity with respect to endogenous RRNPP signalling and its impact on *B. subtilis* development. We wondered whether the App system would enable the import of signalling molecules of exogenous systems. To this end we used the exogenous PlcR-PapR quorum-sensing system from *B. thuringiensis st*. 407 [46, 47]. This RRNPP family quorum-sensing system codes for the PapR signalling peptide ADLPFEF (41 ). Notably, this signal is longer and much more hydrophobic than the endogenous Phr and arbitrium peptides (Fig. S4). These differences may be reflected in differential affinity to the App system. It was previously shown that this system works in the *B. subtilis* lab strain 168 and responds well to either the native peptide or a shorter pentapeptide, LPFEF [48].

**Fig. 8.**
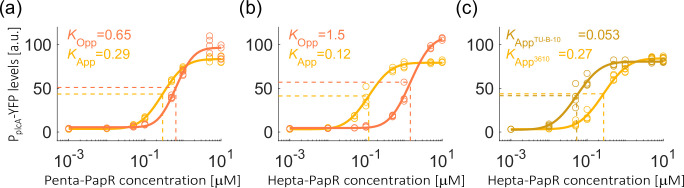
The ability of App-permease systems to import the exogenous signalling peptide PapR. P*_plcA_-*3 ×*yfp* expression levels of the co-cultured PlcR receivers as a function of peptide concentrations. (**a, b**) The response to addition of varying concentrations of PapR peptides to a co-culture of strains expressing either App-only (O^-^;A^+^, AES7075, yellow) and Opp-only (O^+^;A^-^, AES7207, orange). (**a**) Addition of PapR pentapeptide (LPFEF) and (**b**) addition of PapR heptapeptide (ADLPFEF). (**c**) The response of a co-culture of strains App (O^-^;A^+^, AES7075, yellow) and App^TU-B-10T^ (O^+^;A^TU-B-10T^, AES7322, gold) to hepta-PapR (ADLPFEF). Each signaling-peptide concentration was added separately at the same optical density of ~0.1 for cells growing in SMM. YFP levels were measured for single cells using flow cytometry 3 h after the peptide was added. Each dot represents a single biological repeat. Biological repeats at the same concentrations were measured on different days. The data for each strain is fitted to a function of the form $Y=E_{min}+(E_{max}-E_{min})\times erf({log}_{10}(c/K)/n)$ , where c is the concentration of PapR and Y is the YFP expression level. Shown on the graph are best fit results for the half maximal concentration, K , in each experiment.

We integrated the exogenous receptor *plcR*, regulated by a constitutive promoter, and the reporter gene P*_plcA_-*3 ×*yfp* into either App or Opp single-permease expressing strain. Co-culture experiments were performed as described previously with separate addition of the two PapR peptide variants. We find that, in contrast to the Phrs, both permeases allowed for strong uptake of the PapR signal with similar maximal expression levels for the native heptapeptide signal (*E*_max_=109±6 AU for Opp-only and *E*_max_=79±3 for App-only with the native peptide) and the shorter pentapeptide (Fig. 8a, b). The half-maximal expression concentration for the heptapeptide was significantly lower for the App system (*K*=0.12 µM) than for the Opp system (*K*=1.5 µM) (Fig. 8b). This difference was diminished when examining the shorter pentapeptide signal, as the Opp half-maximal concentration was decreased (*K*=0.65 µM), while App’s was increased (*K*=0.29) (Fig. 8a).

To verify that PapR is not imported through a third, uncharacterized, permease, we checked YFP expression levels of a mutant deleted for both permeases (O^-^;A^-^) after adding 10 µM of PapR pentapeptide. This mutant shows YFP levels equal to background levels, which precludes the existence of an additional permease that is capable of transporting the PapR peptide (Fig. S5).

Finally, we examined the ability of a representative App system from the second family of App alleles to transport signalling peptides. To this aim we used the App permease system from *B. subtilis* isolate *spizizenii* TU-B-10T (Fig. 7). Using homologous recombination, we replaced App^3610^ operon with that of *spizizenii* isolate TU-B-10T (Methods), in a background deleted for the Opp operon (O^-^;A^TU-B-10T^). We introduced the *plcR* reporting system into this strain, which was co-cultured with a strain expressing the 3610 App allele (O^-^;A^+^). We then monitored their response as described before for several concentrations of hepta-PapR. We find that both strains showed similar maximal expression levels (*E*_max_=84±2 and *E*_max_=81±3 for App^3610^ and App^TU-B-10T^, respectively) (Fig. 8c). The strains differed in the half-maximal concentration with App^3610^ expressing strain equal to 0.27±0.02 µM; a level similar, though slightly higher than when measured in co-culture with the Opp-only strain. App^TU-B-10T^ expressing strain (O^-^;A^TU-B-10T^) had a five-fold lower half-maximal concentration (0.053±0.004 µM) (Fig. 8c), suggesting that App^TU-B-10^ has a higher affinity than that of App^3610^ for hepta-PapR. Altogether, these results indicate that App allele is able to transport PapR signalling peptides regardless of its lengths, and is superior to Opp, more pronouncedly with the longer form of the PapR peptide.

## Discussion

*Bacillus subtilis* and many related *Bacilli* code for two oligopeptide permease systems, termed Opp and App in *B. subtilis *. The well-studied Opp system has been shown to import signaling-peptides involved in many social behaviours [4, 11, 12, 14, 17–19]. However, due to a frameshift in the *appA* gene of the lab strain 168 and its derivatives [2], less is known about this additional permease system. In this work, we focused on studying the importance of the Opp and the two App variants to the different signalling systems and the resulting developmental consequences. We chose to do this in the ancestral wild-type strain of the lab strain, as it does not have the frameshift mutation at the App gene, and it shows strong biofilm formation. We used the variant which lacks the large pBS32 plasmid, as this plasmid has been previously shown to repress competence in a signalling independent manner [35].

Our results demonstrate that the App system is not well-suited for the import of native RRNPP signalling peptides and correspondingly, have only a minor effect on many of the related phenotypes, including sporulation, surfactin enzymes expression, biofilm formation and phage infection. In contrast, our results suggest that App is superior to Opp in the uptake of the PapR heptamer and as effective in the uptake of the shorter pentapeptide form of PapR. Notably, peptide uptake by oligopeptide permeases can have a dramatic effect on the extracellular concentration of the peptides [39, 40], and therefore it may be that App impact on PlcR signalling has an effect on the behaviour of *B. cereus* group species in mixed communities of *B. subtilis* group and *B. cereus* group species.

With respect to sporulation, previous analysis suggested that Opp deletion in the lab strain JH642 led to a large reduction in sporulation efficiency, which was complemented by restoring the function of the App system in this strain [2]. In contrast, we found that both permease systems had only a small effect on sporulation in the biofilm-forming strain 3610. This seeming contradiction may stem from a reduced activity of RapA, the major known Rap-Phr system affecting sporulation in the biofilm strain. RapA expression is regulated by ComA and we have previously shown that ComA activity is lower in the biofilm-forming strain we used in this work [31, 49].

It has been suggested that App specificity might be skewed towards longer peptides [50], which agrees well with its effectiveness in importing the PapR heptapeptide. App was also as effective as Opp in uptaking the PapR pentapeptide, but not in importing the PhrC/F/P pentapeptides. Previous work has identified several App transported peptides, including the nonapeptide RPPGFSPFR [2, 50]. Another work observed that App was able to transport the tetra and the pentapeptides, FGFG and FLEEI [2]. Here we found that App strongly imports the PapR variants [AD]LPFEF. Altogether these results suggest that the uptake of App permease is dependent on the oligopeptides' composition as much as on length. The data indicates a possible advantage for phenylalanine-containing peptides. This may stem from the high hydrophobicity of these peptides (Fig. S4). The Opp system is more likely to be a universal transporter of somewhat shorter peptides. Oligopeptides in *B. subtilis* has been implied to play a role in nutrition acquisition [51], osmoprotection [52], sensitivity to toxins [53] and signalling. Yet, there is no known specific role for hydrophobic peptides in *Bacillus subtilis* physiology and the hydrophobicity hypothesis is therefore not further supported by a specific biological function.

Selection for hydrophobic residues has been shown to occur in other Opp homologues. For example, the plasmid pCF10 of *Enterococcus faecalis *, codes for the Prg RRNPP quorum-sensing system, which controls its conjugation. This plasmid also expresses PrgZ, a OppA homolog, which has a much higher specificity to the hydrophobic signalling peptides of the Prg system than the native OppA homologue of the bacterium [54].

The notion that App has a signalling independent function is further strengthened by its unique phylogeny. While Opp phylogeny follows the general housekeeping phylogeny, our analysis shows that the App system and specifically the *appAB* genes have two divergent alleles, which were moved by horizontal gene transfer (most likely more than once) within the *B. subtilis* group. This seems to have occurred through recombination over the *appC* gene and at the end of the *app* operon. Such recombination can occur through transformation or by other means and have been shown to occur in other systems such as the ComQXP quorum-sensing system [55]. Such horizontal gene transfer of core genes may suggest that the function of these genes is under diversifying evolutionary selection [55]. From a molecular standpoint *appA* codes for the peptide-binding protein, while *appB* is probably in direct interaction with it, making the change in this pair sufficient. Our experimental analysis of the two App allelic variants suggests that they are more similar to each other than to the Opp system, but still differ in their affinity to the PapR peptide (Fig. 8). The selective pressure that leads to the formation and horizontal transfer of the two *appAB* alleles is most likely unrelated to signalling and remains unknown.

Interestingly, recent analysis identified two functioning Oligopeptide permease systems in *B. thuringiensis st*. 407, from which the PapR system was cloned [28]. In contrast to our findings, it was found that both systems are strongly involved in RRNPP signalling [28]. In agreement with our results, this suggests that there is a pressure for the diversification of the oligopeptide permease system throughout the *Bacillus genus*, but that the effect of this permease diversity on signalling may vary between species.

## Supplementary Data

Supplementary material 1
