# Supplementary material for: Major distinctions between the two oligopeptide permease systems of Bacillus subtilis with respect to signaling, development and evolutionary divergence
Source: Microbiology (Reading). 2023 Sep 27;169(9):001382. doi: 10.1099/mic.0.001382 (PMC10569065; doi:10.1099/mic.0.001382)
Supplement: Supplementary material 1 [file mic-169-1382-s001.pdf]

Supplementary figures

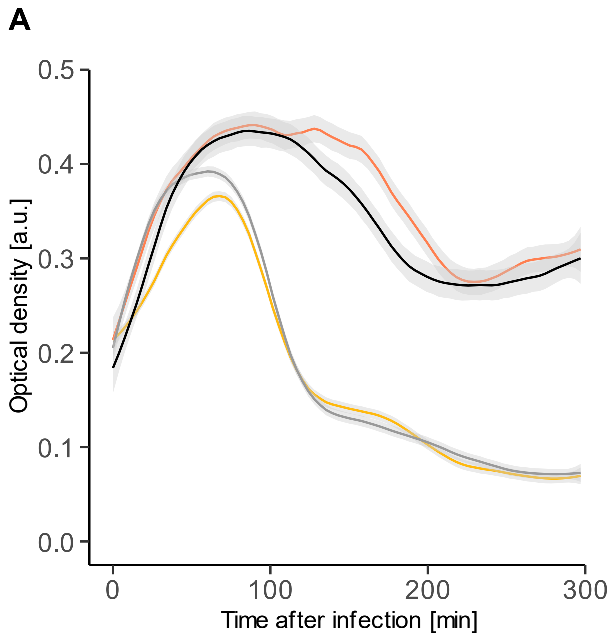

Figure S1| The impact of oligopeptide permeases on phage infection in the presence of an arbitrium lysogenization signal. Growth curves of different variants of *B. subtilis* 3610 plasmid-free strain: A wildtype expressing App and Opp (O^+;A^+, DS2569, black), Opp-only strain (O^+;A^-, AES7144, orange) with full deletion of *app* operon, App-only strain (O^-;A^+, AES6372, yellow) with full deletion of *opp* operon and a mutant deleted in both operon (O^-;A^-, AES7249, gray) (see legend). All infected with ϕ3T at MOI = 0.1 in LB in the presence of 10 µM of the synthetic peptide SAIRGA. Growth curves are represented as the mean optical density (dark color) and its error (lighter shade) as a function of time (minutes) taken using a plate-reader. Arbitrium peptide was added at time 0 at an OD_{600}=0.3. Results are the average of three technical repeats. These are two biological repeats for Figure 4 performed in independent days.

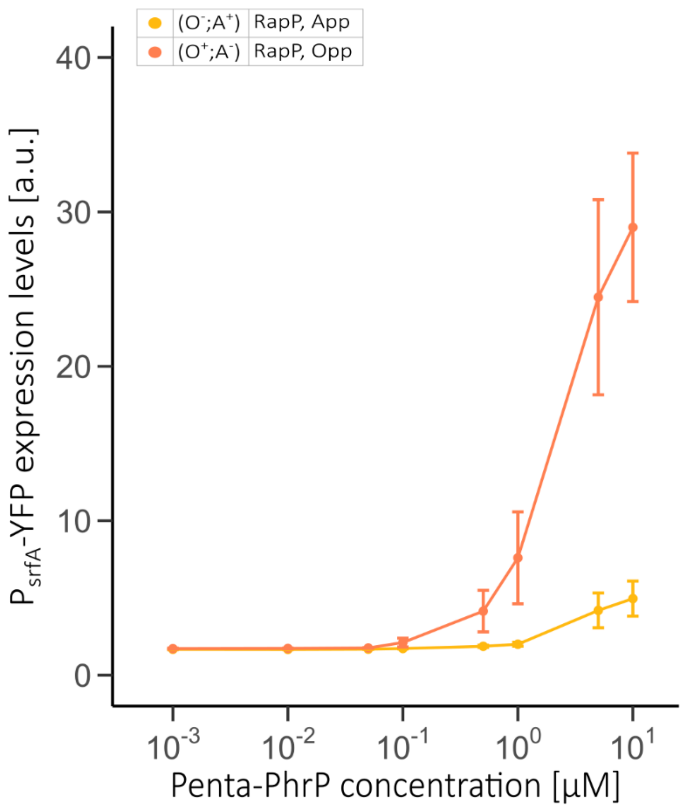

Figure S2| App expressing strain does not respond to the extracellularly added penta PhrP. The expression levels of P_{srfA}-3×*yfp* as a function of pentapeptide (DRAAT) concentrations for strains expressing the RapP only. Each strain either expresses App-only (O^-;A^+, AES5514) or Opp-only (O^+;A^-, AES5524) in yellow and orange, respectively. Each concentration was added separately at the same optical density of ~0.1 for cells growing in SMM. YFP levels were measured for single cells using flow cytometry three hours after the peptide was added to App and Opp co-culture. Each dot represents the mean (±s.e.m) of at least three independent biological repeats measured in different days.

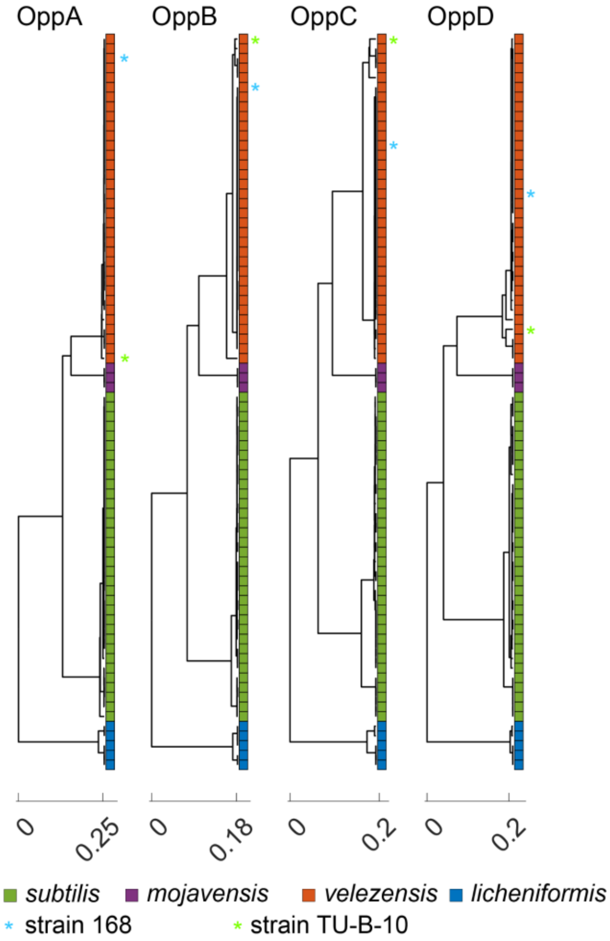

Figure S3| Opp phylogeny corresponds to the species phylogeny. Shown are the phylogenetic tree of the proteins of OppA-D for different strains belonging to the species *B. subtilis* (green), *B. mojavensis* (purple), *B. velezensis/B. amylolyquefaciens* (orange), *B. licheniformis* (blue). As can be seen, strains from the same species are all monophyletic. Opp proteins of strain 3610 and of strain TU-B-10 are shown in cyan and light green correspondingly. contrast with Fig. 7 of the main manuscript.

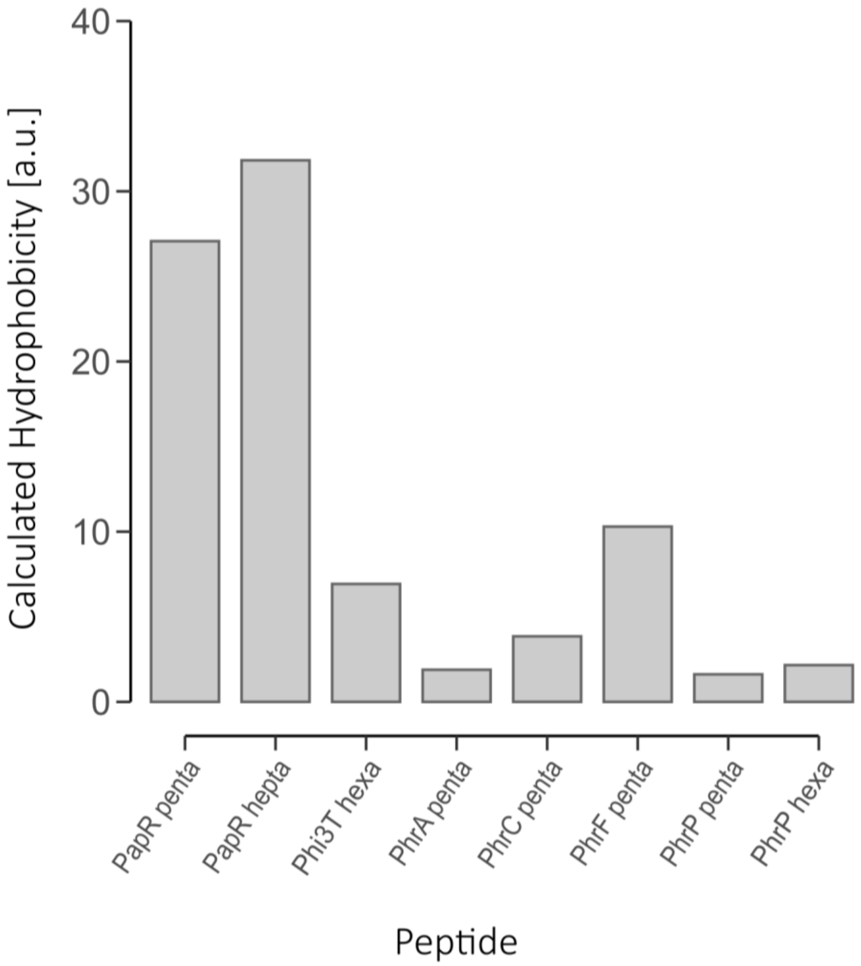

Figure S4| Calculated hydrophobicity for signaling peptides. The calculated hydrophobicity for several signaling peptides using Peptide Synthesis and Proteotypic Peptide Analyzing Tool of thermo fisher scientific. https://www.thermofisher.com/il/en/home/life-science/protein-biology/peptides-proteins/custom-peptide-synthesis-services/peptide-analyzing-tool.html

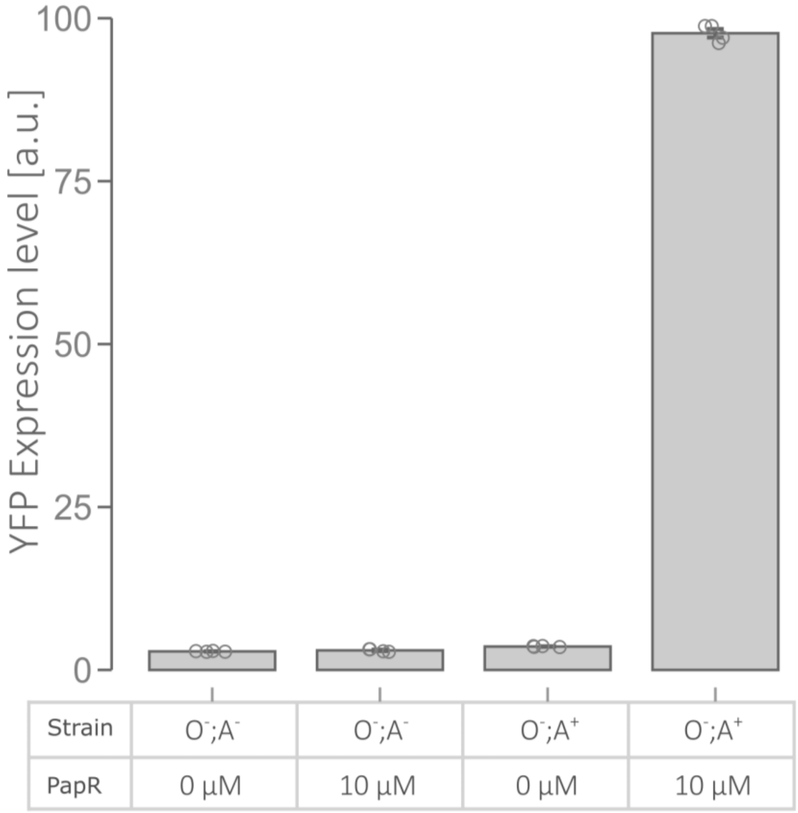

Figure S5| A mutant in App and Opp does not respond to the extracellularly added hepta PapR. Expression levels of the reporter P_{plcA}-3×*yfp* either in the presence or absence of PapR Heptapeptide (ADLPFEF), for two strains. Left columns: a mutant deleted in both App and Opp operons (O^-;A^-, AES7075), right columns: a strain expressing App only (O^-;A^+, AES8170). 10 µM was at the same optical density of ~0.1 for cells growing in SMM as monoculture. YFP levels were measured for single cells using flow cytometry three hours after the peptide was added. Bars represent the mean (±s.e.m) of at least three independent biological repeats.
